# New Gene Markers Expressed in Porcine Oviductal Epithelial Cells Cultured Primary In Vitro Are Involved in Ontological Groups Representing Physiological Processes of Porcine Oocytes

**DOI:** 10.3390/ijms22042082

**Published:** 2021-02-19

**Authors:** Magdalena Kulus, Wiesława Kranc, Katarzyna Wojtanowicz-Markiewicz, Piotr Celichowski, Agata Światły-Błaszkiewicz, Eliza Matuszewska, Patrycja Sujka-Kordowska, Aneta Konwerska, Maciej Zdun, Rut Bryl, Maria Wieczorkiewicz, Jakub Kulus, Bogusława Stelmach, Katarzyna Stefańska, Joanna Budna-Tukan, James N. Petitte, Paul Mozdziak, Kornel Ratajczak, Jan Matysiak, Jędrzej M. Jaśkowski, Michał Nowicki, Bartosz Kempisty

**Affiliations:** 1 Department of Veterinary Surgery, Institute of Veterinary Medicine, Nicolaus Copernicus University in Torun, 87-100 Torun, Poland; magdalena.kulus@umk.pl (M.K.); kasiawojtanowicz@gmail.com (K.W.-M.); kornel@umk.pl (K.R.); 2 Department of Anatomy, Poznan University of Medical Sciences, 60-781 Poznan, Poland; wkranc@ump.edu.pl (W.K.); rutbryl@gmail.com (R.B.); 3 Department of Histology and Embryology, Poznan University of Medical Sciences, 60-781 Poznan, Poland; pcelichowski@ump.edu.pl (P.C.); psujka@ump.edu.pl (P.S.-K.); akonwer@ump.edu.pl (A.K.); k.stefanska94@o2.pl (K.S.); jbudna@ump.edu.pl (J.B.-T.); mnowicki@ump.edu.pl (M.N.); 4 Department of Inorganic and Analytical Chemistry, Poznan University of Medical Sciences, 60-780 Poznan, Poland; aswiatly@ump.edu.pl (A.Ś.-B.); eliza.matuszewska@ump.edu.pl (E.M.); jmatysiak@ump.edu.pl (J.M.); 5 Department of Anatomy and Histology, University of Zielona Gora, 65-046 Zielona Gora, Poland; 6 Department of Basic and Preclinical Sciences, Institute of Veterinary Medicine, Nicolaus Copernicus University in Torun, 87-100 Torun, Poland; maciejzdun@umk.pl (M.Z.); maria.wieczorkiewicz@umk.pl (M.W.); 7 Department of Diagnostics and Clinical Sciences, Institute of Veterinary Medicine, Nicolaus Copernicus University in Torun, 87-100 Torun, Poland; jakub.kulus@umk.pl (J.K.); jmjaskowski@umk.pl (J.M.J.); 8 Division of Infertility and Reproductive Endocrinology, Department of Gynecology, Obstetrics and Gynecological Oncology, Poznan University of Medical Sciences, 60-535 Poznan, Poland; b_stelmach@wp.pl; 9 Prestage Department of Poultry Science, North Carolina State University, Raleigh, NC 27695, USA; jnppo@ncsu.edu (J.N.P.); pemozdzi@ncsu.edu (P.M.)

**Keywords:** pig, epithelial cells, microarray assays, long-term in vitro culture, oviduct, transcriptomics, proteomics

## Abstract

Changes that occur within oviducts after fertilization are dependent on post-ovulation events, including oocyte-oviduct interactions. Although general processes are well-defined, the molecular basis are poorly understood. Recently, new marker genes involved in ‘cell development’, ‘cell growth’, ‘cell differentiation’ and ‘cell maturation’ processes have been identified in porcine oocytes. The aim of the study was to assess the expression profile of genes in primary in vitro cultured oviductal epithelial cells (OECs), clustered in Gene Ontology groups which enveloped markers also identified in porcine oocytes. OECs (from 45 gilts) were surgically removed and cultured in vitro for ≤ 30 days, and then subjected to molecular analyses. The transcriptomic and proteomic profiles of cells cultured during 7, 15 and 30 days were investigated. Additionally, morphological/histochemical analyzes were performed. The results of genes expression profiles were validated after using RT-qPCR. The results showed a significant upregulation of *UNC45B, NOX4, VLDLR, ITGB3, FMOD, SGCE, COL1A2, LOX, LIPG, THY1* and downregulation of *SERPINB2, CD274, TXNIP, CELA1, DDX60, CRABP2, SLC5A1, IDO1, ANPEP, FST*. Detailed knowledge of the molecular pathways occurring in the OECs and the gametes that contact them may contribute both to developments of basic science of physiology, and new possibilities in advanced biotechnology of assisted reproduction.

## 1. Introduction

Cells of the reproductive tract undergo substantial morphological and biochemical modification in mammals. These changes are regulated by ovulation, embryo-maternal interaction and proper embryo implantation in uterine endometrium [1]. Furthermore, the changes that occur within oviducts and/or the uterus are highly dependent on the post-ovulation events, including oocyte-oviduct interactions and embryo-endometrial ‘dialog’ after successful fertilization [2]. 

Changes within the oviductal epithelium are induced by oocyte-maternal tissue interactions during gamete transfer [3]. Recently, it was observed that oviductal tissue, including epithelium, may be cultured in vitro for short and long-term periods [4]. In oviductal epithelial cell (OEC) in vitro culture, cells may change their morphology and biochemical properties [5,6]. The biochemical and molecular changes involve modifications of gene and protein expression profiles that may regulate gamete-OEC interactions [2]. This unique ‘dialog’ is one of the processes that may significantly influence successful fertilization in vivo. While the in vivo events leading to fertilization are difficult to investigate, in vitro co-culture systems and/or 3D cultivation models may provide new opportunities in research of this topic. 

While it was well documented in the case of human ovarian granulosa cells, which may differentiate into several other, even distinct cell types, such as chondroblasts, osteoblasts and/or adipocytes [7], the process of OEC in vitro differentiation during long-term primary culture is still not entirely understood. It was determined in several cell type models that stemness specificity and/or pluripotency of cells may be defined by their cellular development, proliferation and differentiation capability [8]. Other sources state that different cells might achieve stem-like characteristics during long-term primary in vitro culture. Most of the cells collected from adult organs have some potential for growth and proliferation in vitro, which are primary features characterizing their potency for application in regenerative and reconstructive medicine [9].

Therefore, in the present study, the expression profile of genes clustered in Gene Ontology (GO) groups, which included ‘cell development’, ‘cell growth’, ‘cell differentiation’ and ‘cell maturation’, was assessed. Moreover, new processes orchestrated by OECs during in vitro culture, as well as potential markers of OEC growth were characterized. Lastly, the signaling pathways regulating stem cell pluripotency in porcine OEC long-term cultures were evaluated.

## 2. Results

### 2.1. Whole Transcriptome Profiling

In this study, we performed analysis of gene expression changes from porcine OECs by whole transcriptome profiling using Affymetrix microarrays. Samples from days 7, 15 and 30 of in vitro culture were evaluated. Affymetrix Porcine Gene 1.1 ST Array Strip was used and expression of 12,257 transcripts was obtained. Genes with fold change >|2| and a corrected *p* < 0.05 were considered as differentially expressed. This set of genes consisted of 2533 different transcripts, with the complete list of individual gene expression values available in the GEO database (https://www.ncbi.nlm.nih.gov/geo/query/acc.cgi?acc=GSE100190).

### 2.2. GO BP Terms and KEGG Pathway Analysis

**Figure 1:**
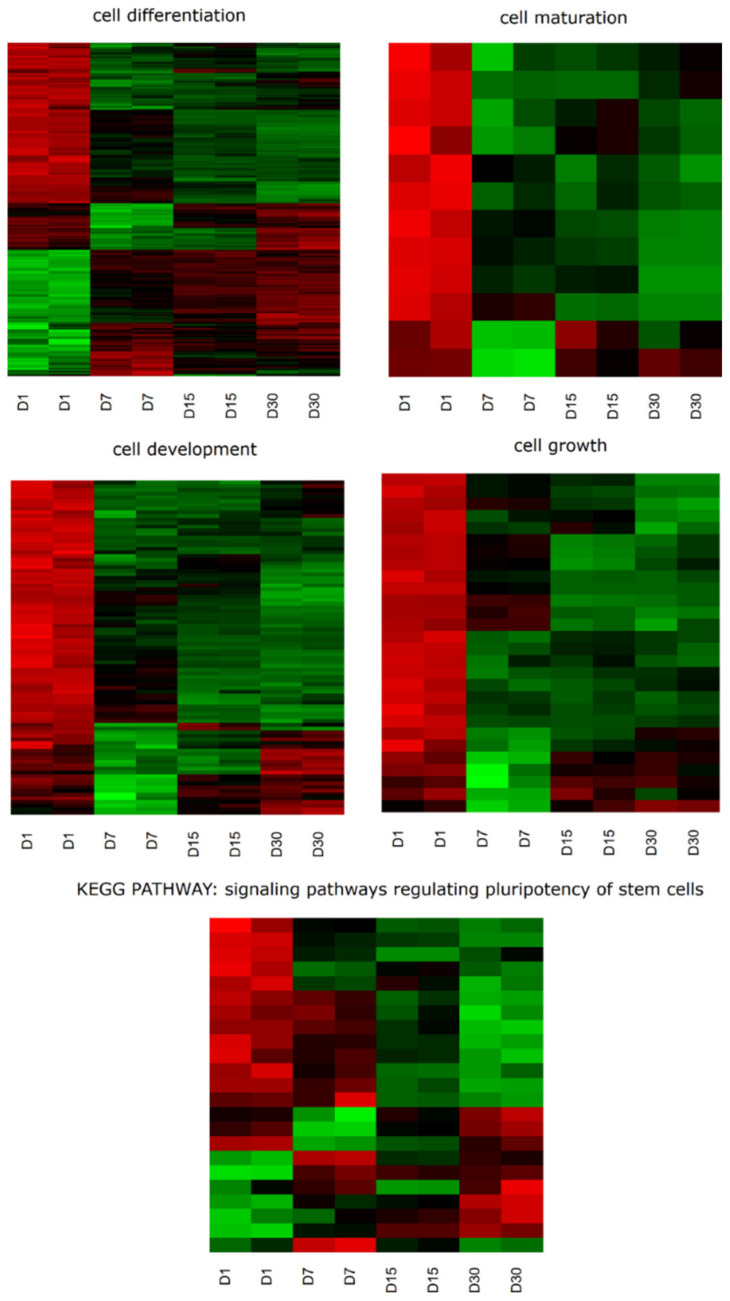
Heatmap representation of differentially expressed genes belonging to the ‘cell differentiation’, ‘cell growth’, ‘cell maturation’ and ‘cell development’ Gene Ontology biological process terms, as well as the ‘signaling pathways regulating pluripotency of stem cells’ KEGG pathway. The results obtained from microarray analysis are presented by a color signal intensity scale (green-upregulation; red-downregulation). The signal intensity log2 values for each individual gene were changed to a Row Z-Score scale, ranging from −2, minimum expression to +2, maximum expression for a particular gene. KEGG = Kyoto Encyclopedia of Genes and Genomes; D, day of culture.

DAVID software was used for extraction of the GO BP terms that contain differently expressed transcripts. Up and downregulated gene sets were screened separately and only gene sets where adjusted *p* < 0.05 were selected. DAVID software analysis suggested that differently expressed genes of interest belonged to 657 GO terms. The present study focused on 369 genes belonging to ‘cell differentiation’, ‘cell growth’, ‘cell maturation’ and ‘cell development’ terms, as well as ‘signaling pathways regulating pluripotency of stem cells’ KEGG pathway. Overall, 133 of these genes were downregulated and 236 were upregulated. These genes were subjected to hierarchical clustering and are presented as heatmaps (Figure 1).

In the present study, the transcriptional profile of OECs at different periods of in vitro culture time (1, 7, 15, 30 days) were analyzed. Using a microarray approach, the role of genes in important cell life processes, such as ‘cell development’, ‘cell growth’, ‘cell differentiation’ and ‘cell maturation’ during long-term in vitro primary culture of OECs were examined. Gene expression values in particular GO groups were analyzed. In the ‘cell differentiation’ group, >50% of the genes were downregulated on the first day of culture. On the following days of culture (7, 15, 30), the expression of these particular genes was reversed, showing upregulation. Similarly, all of the genes belonging to the ‘cell maturation’ group were downregulated on the first day of culture, then most of them were upregulated between day 7 and 30. These results coincided with expression in the ‘cell development’ and ‘cell growth’ groups. However, in the case of ‘signaling pathways regulating pluripotency of stem cells’, the first day was dominated by downregulated genes, with trend reversal occurring on day 30. On days 7 and 15, genes from this group showed varied expression (Figure 1).

**Figure 2:**
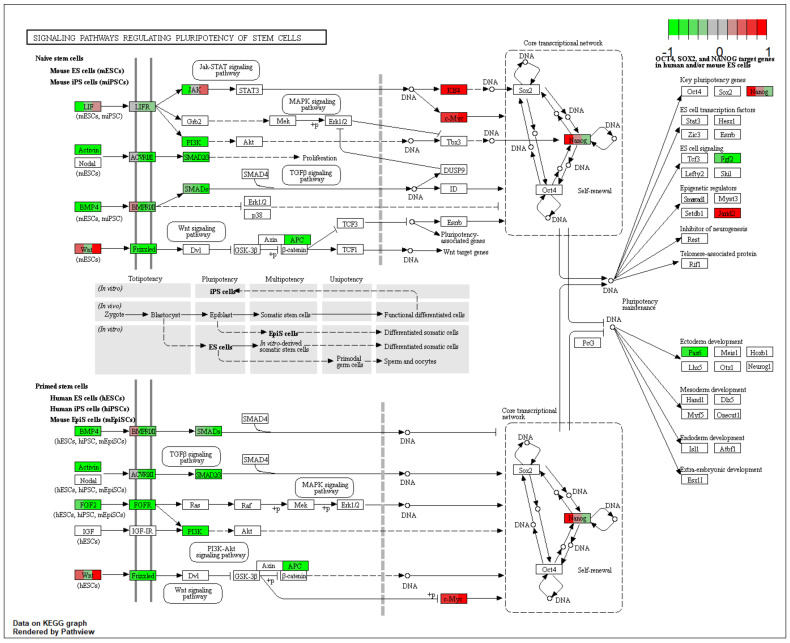
‘Signaling pathways regulating pluripotency of stem cells’ KEGG pathway. Arbitrary signal intensity acquired from microarray analysis is represented by colors (green, higher; red, lower expression). Each gene of interest is assigned three colors, each representing change in expression levels between D7/D1, D15/D1 and D30/D1, respectively. KEGG, Kyoto Encyclopedia of Genes and Genomes; D, day of culture. Copyright permission obtained.

The gene symbols, fold changes in expression, Entrez gene identification numbers and corrected *p*-values of these genes are shown in Table 1. The differently expressed genes from ‘signaling pathways regulating pluripotency of stem cells’ KEGG pathway were marked on the pathway and are presented in Figure 2.

**Figure 3:**
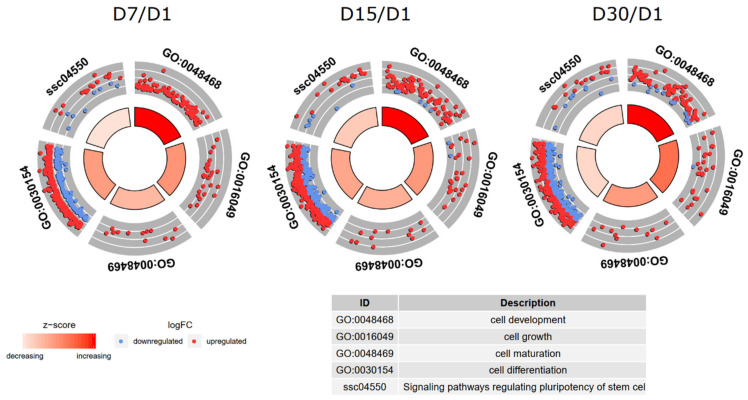
Circle plot showing the differently expressed genes and z-score ‘cell differentiation’, ‘cell growth’, ‘cell maturation’ and ‘cell development’ GO BP terms, as well as ‘signaling pathways regulating pluripotency of stem cells’ Kyoto Encyclopedia of Genes and Genomes pathway. The outer circle is a scatter plot, showing fold changes of every investigated gene of the given GO. The colored dots show the decrease (blue) and increase (red) of regulation. The central columns display the z-score of every GO BP and the width of every bar expresses to the amount of genes within GO BP term. GO, Gene Ontology; BP, biological process; FC, fold change; D, day of culture.

The enrichment of each GO BP term, as well KEGG pathway were calculated as z-score and shown on the circle diagram (Figure 3).

**Figure 4:**
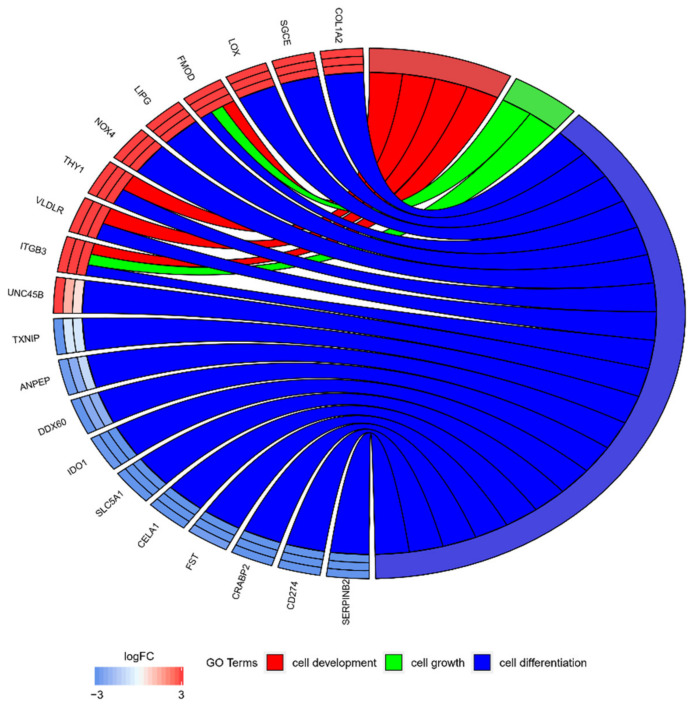
The representation of the mutual relationship 10 most upregulated and 10 most downregulated genes that belongs to the “cell differentiation”, “cell growth”, “cell maturation” and “cell development” GO BP terms as well as “signaling pathways regulating pluripotency of stem cells” KEGG pathway. The bands represent what gene was included in exactly which ontology group. LogFC (logarithm from fold change) between each culture day (7/1; 15/1; 30/1) respectively is shown as the middle circle. The order of genes presented was selected based on logFC and represents from strongest to weakest changed.

**Figure 5:**
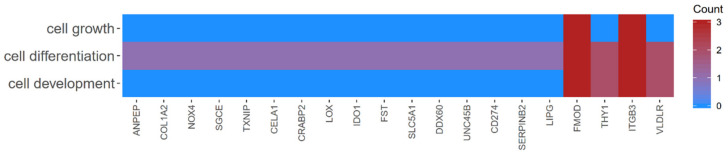
Heatmap showing the gene occurrence between 10 most upregulated and 10 most downregulated genes that belongs to the “cell differentiation”, “cell growth”, “cell maturation” and “cell development” GO BP terms as well as “signaling pathways regulating pluripotency of stem cells” KEGG pathway. The red color is associated with gene occurrence in the GO Term. The intensity of the color is corresponding to amount of GO BP terms that each gene belongs to.

Among the 369 genes, the 10 most upregulated (*COL1A2, SGCE, LOX, FMOD, LIPG, NOK4, THY1, VLDLR, ITGB3, UNC45B*) and 10 most downregulated (*TXNIP, ANPEP, DDX60, IDO1, SLC5A1, CELA1, FST, CRABP2, CD274, SERPINB2*) were selected for further analysis. Since any given gene can belong to multiple GO groups, gene intersections between selected GO BP terms were examined. The relation between analyzed GO BP terms was presented as a circle plot (Figure 4), as well as a heatmap (Figure 5), which demonstrated that all of the 20 selected genes belong to the “cell differentiation” ontology group. In addition, the *THY1* and *VLDLR* genes represent the “cell development” of GO and the *ITGB3* and *FMOD* genes belong to the “cell growth” ontology group.

Most of the genes presented in the present study only belong to the ‘cell differentiation’ group (Figure 5). However, *THY1* and very low-density lipoprotein receptor (*VLDLR*) each belong to two GO groups, ‘cell differentiation’ and ‘cell development’. Fibromodulin (*FMOD*) and integrin subunit β-3 (*ITGB3*) are members of all three of the analyzed GO groups. This distribution suggests that *THY1* and *VLDLR* may serve as differentiation markers. A total of four genes, namely *ITGB3*, *FMOD*, *VLDLR* and *THY1*, were associated with the ‘cell development’ term. As these genes were upregulated, these can be associated with strong potential for OEC development during long-term culture. Two of these genes also belong to ‘cell growth’, *ITGB3* and *FMOD* (Figure 5).

A group of genes that are involved in different metabolic processes were downregulated after in vitro culture (7, 15 and 30 days), compared to first day of its course. These included *SERPINB2*, thioredoxin-interacting protein (*TXNIP*), cellular retinoic acid binding protein 2 (*CRABP2*), solute carrier family 5 member 1 (*SLC5A1*), alanyl aminopeptidase (*ANPEP*) and follistatin (*FST*; Figure 4).

### 2.3. Interaction Network Analyses

**Figure 6:**
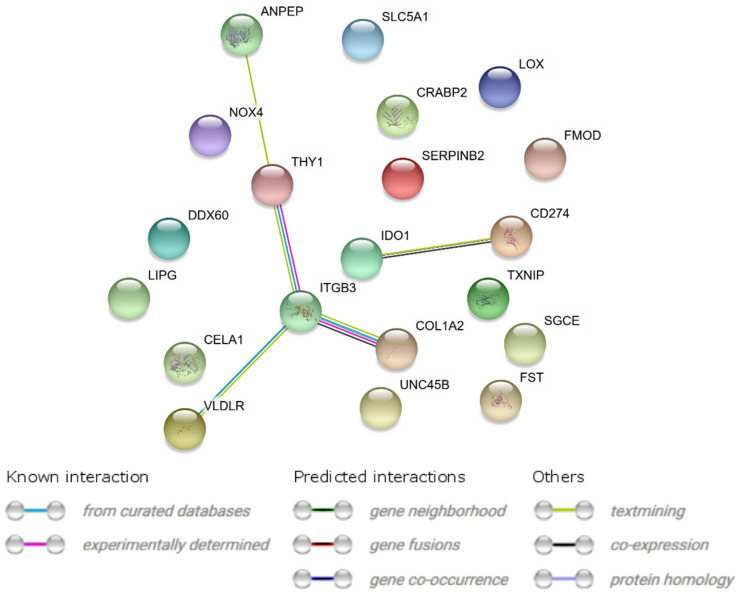
STRING-generated interaction network 10 most upregulated and 10 most downregulated genes that belongs to the “cell differentiation”, “cell growth”, “cell maturation” and “cell development” GO BP terms as well as “signaling pathways regulating pluripotency of stem cells” KEGG pathway. The intensity of the edges reflects the strength of interaction score.

**Figure 7:**
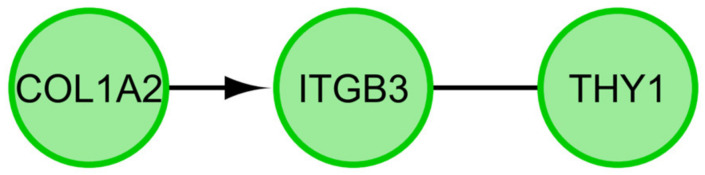
Functional interaction (FI) between 10 most upregulated and 10 most downregulated genes that belongs to the “cell differentiation”, “cell growth”, “cell maturation” and “cell development” GO BP terms as well as “signaling pathways regulating pluripotency of stem cells” KEGG pathway. In following figure “->“ stands for activating/catalyzing, “-” for FIs extracted from complexes or inputs.

A STRING interaction network was generated among differentially expressed genes belonging to each of the selected GO BP terms (Figure 6). Co-expression was only demonstrated in two cases, in the *ITGB3* and *COL1A2* genes, and in the *IDO1* and *CD274* genes. Overall, 13 of the analyzed genes did not show direct interactions with other genes of interest (Figure 6). Lastly, the FIs between chosen genes were examined (Figure 7) using the REACTOME FIViz application in Cytoscape 3.6.0 software. By analyzing selected genes, these results demonstrated the functional interaction between the *COL1A2* and *ITGB3* genes. Additionally, *THY1* was shown to be extracted from complexes or inputs to *ITGB3*.

### 2.4. Real-Time Quantitative Polymerase Chain Reaction (RT-qPCR) Analysis

**Figure 8:**
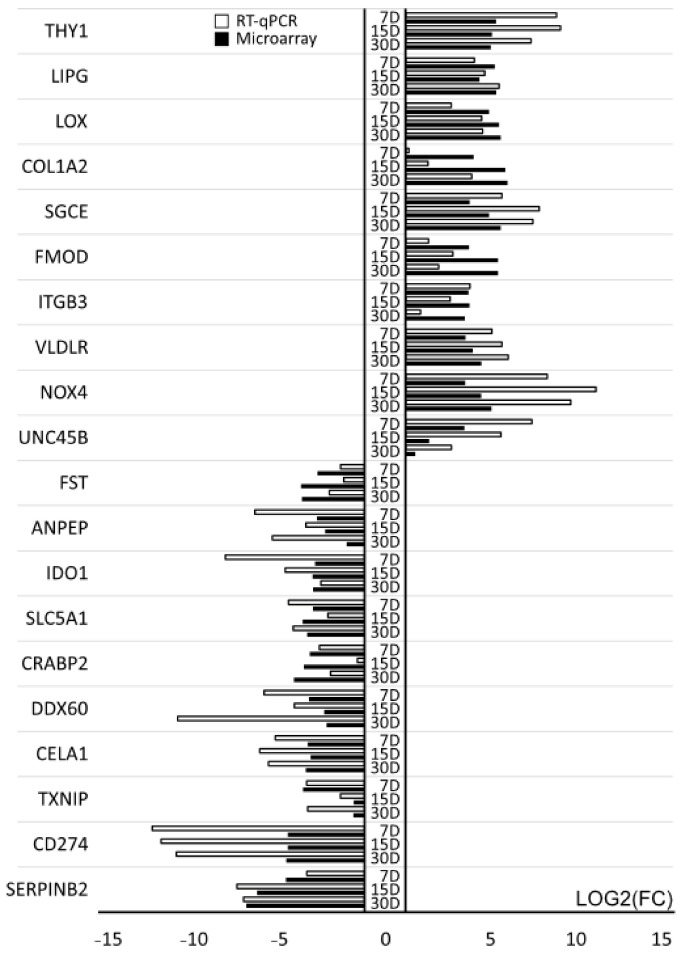
RT-qPCR quantitative validation of microarray results presented in a form of a bar graph.

The RT-qPCR method was used to validate 20 genes selected during the microarray analysis. The results obtained during both the microarray and the RT-qPCR are presented in the bar graph. The goal of RT-qPCR was to validate the direction of change in expression of individual genes. The scale of differences in levels of expression differed between the two methods (Figure 8).

### 2.5. Nano LC-MALDI-TOF/TOF MS/MS Analysis

**Figure 9:**
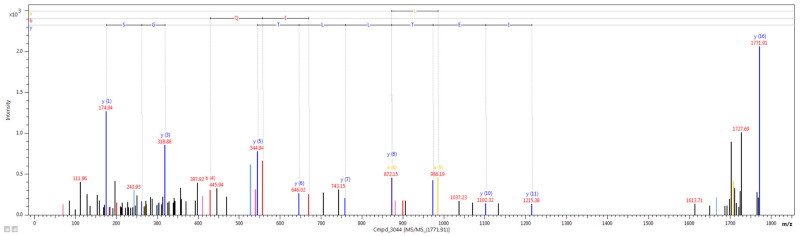
The representative example of the mass spectrum of *m*/*z* 1417.6834, identified as one of the COL1A2 peptides.

The nano LC-MALDI-TOF/TOF MS/MS analysis allowed for identification of 3240 unique tryptic peptides, which corresponded to 389 proteins. The representative mass spectrum (*m*/*z* 1417.6834), which was identified as one of the collagen type I α 2 chain (COL1A2) peptides is shown in Figure 9.

### 2.6. Morphology of OECs

**Figure 10:**
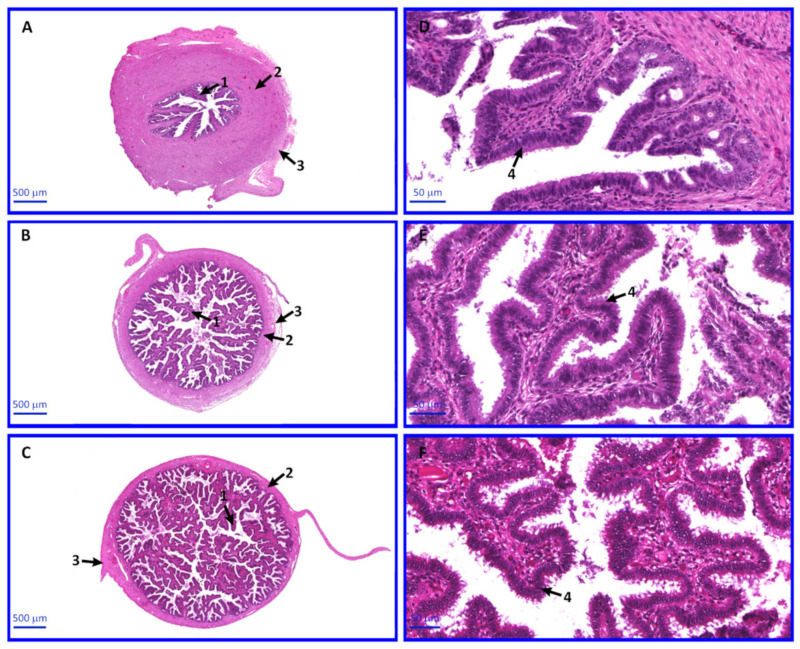
Photomicrograph representing particular segments of porcine oviduct. (**A**)–the isthmus, (**B**)–the ampulla, (**C**)–the infundibulum, (**D**–**F**)–mucosal folds lined with simple columnar ciliated epithelium. Arrows: 1–mucosal folds, 2–muscular layer, 3–serosa, 4–simple columnar ciliated epithelium.

Light microscope observations of sections stained with the H&E method revealed a normal morphological structure of particular parts of oviducts. Mucosal folds of the isthmus are poorly developed, while the smooth muscles form a thick layer. In the ampulla, muscular layer is thinner, and mucosal folds are branched, numerous and longer compared with that in the isthmus. The mucosa in the infundibulum forms long, finger-like folds, while the muscular layer is very thin. The mucosa in each segments of oviducts is lined with simple columnar ciliated epithelium (Figure 10).

The oviduct fragments were collected for morphological analysis of organs, and specific parts of the oviducts were assessed. The aim was to confirm the morphological differentiation of the specific oviduct sections and to exclude potential pathological changes in the epithelium that could affect the expression of analyzed genes. As a result of this subsection, correct morphological structure of individual parts of the fallopian tube was found, no pathologies or structural disorders were observed.

**Figure 11:**
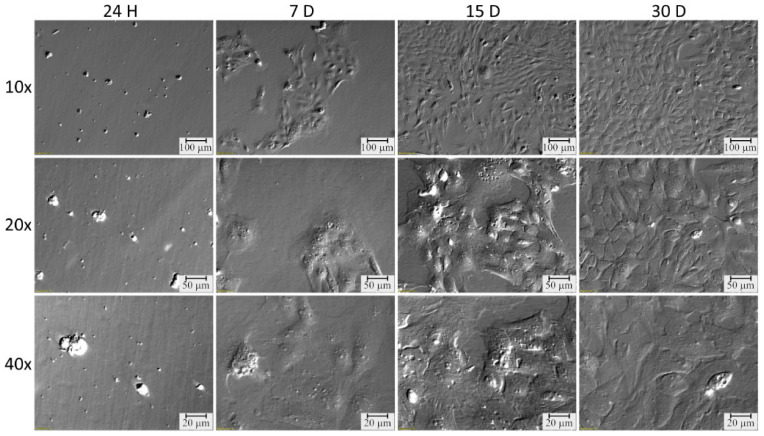
Pictures of porcine oviduct epithelial cells cultures used for the experiment, presenting changes in their morphology, following 24 h, and 7, 15 and 30 days of in vitro culture (magnification ×10, 20 and 40, as indicated).

Meanwhile, changes in cell morphology were monitored throughout the in vitro primary cultivation conducted in this study. Numerous cultivations were conducted simultaneously, most of which showed comparative changes in morphology, allowing the OECs to be considered suitable for the study. OECs underwent extensively morphological changes over the duration of cultivation. From star-like cells, OECs turned into wider cells, taking on a fibroblast-like shape over time. Pictures taken under an inverted microscope (relief contrast) show the dynamics of morphological changes observed during in vitro culture of OECs (Figure 11).

## 3. Discussion

The process of successful fertilization depends on a number of factors that facilitate complex physiological and biochemical processes, as well as cellular interactions [10,11]. The processes of morphological and biochemical changes occurring in the fallopian tubes during oocytes transport and subsequent fertilization are generally known, but their molecular background is still unclear [11]. These changes particularly concern the oviduct epithelial cells (OECs), having effect on proper oocyte transport and fertilization. The mucous membrane of the oviduct is strongly folded and consists of a ciliated simple columnar epithelium (responsible for the movement of oocytes and sperm) and glandular cells, which produce mucus [12,13]. Furthermore, the hormones produced in the ovary (oestrogen and progesterone) during the oestrous cycle were found to affect the transciptome of the oviduct in pigs [14,15]. It is likely that the regulation of processes occurring in female reproductive organs takes place at the level of direct contacts and intercellular signals, which are initiated by steroid hormones. Lee et al. [16] demonstrated that co-culture of canine oviductal cells with porcine oocyte-cumulus complexes, coupled with progesterone stimulation, induced the expression of epidermal growth factor-like proteins. Thus, oviductal cells effectively improved the development of porcine oocyte-cumulus complexes and subsequent embryo development. Nevertheless, even though in vitro cell cultures offer great opportunities to explore underlying mechanisms taking place in and between cells [17], the complexity of regulating important life processes is very high. Therefore, it was important to evaluate the gene expression profile of in vitro culture of porcine OECs.

It is important to evaluate the expression of genes potentially involved in the processes of maturation, development, growth and differentiation of oviductal epithelial cells in later stages of in vitro cultivation. The focus was on genes related to the ontology group: “cell differentiation”, “cell growth” and “cell development”, because the expression of these groups of genes, of which has shown changes during in vitro cultivation (IVC), indirectly affects the assessment of the competence of OECs in their relationship with gametes and their subsequent development.

All the genes analyzed showed membership in the “cell differentiation” ontology group. The majority (16) of the selected 20 genes showed belonging to this group only. The genes representing the “cell development”, “cell growth” and “cell differentiation” groups were *ITGB3* and *FMOD*, which showed up-regulation of expression.

The first gene belonging to all the aforementioned GO terms is *ITGB3*, also known as *CD61*. *ITGB3* transcripts have primarily been detected in porcine blood platelets and hematopoietic tissues. Integrins are a group of adhesion molecules involved in important cellular processes [18]. Previous studies have shown a link between *ITGB3* expression and infection of porcine cells by classical swine fever virus strains [19,20]. Increased expression of this gene was also observed in porcine ovarian granulosa cells during in vitro culture, where ITGB3 was shown to be involved in differentiation, development and morphogenesis [21,22]. It appears that there was an upregulation of *ITGB3* expression after in vitro culture, compared with the first day of cultivation.

The second upregulated gene, also belonging to all three ontological groups, is *FMOD*. It is a protein-coding gene expressed, amongst others, in porcine ovary and subcutaneous adipose tissue [23]. It has been suggested that *FMOD* was one of the cell surface molecules that could serve as a marker for a human mesenchymal stem cell (MSC) subpopulation with limited differentiation potential [24]. *FMOD* strongly promotes angiogenesis in various models of skin wounds, suggesting an important role in tissue healing [25]. The expression of this gene in relation to the porcine reproductive system was described in gene expression profile studies of porcine granulosa cells during in vitro culture [22]. Thus, *FMOD* could be regarded as a potential marker of MSC proliferation and differentiation.

Genes showing membership in two ontology groups (“cell development” and “cell differentiation”) were *VLDLR* and *THY1*. *VLDLR*, the multifunctional receptor [26], is ubiquitously expressed in porcine ovary, heart and other tissues [23]. In the present study, *VLDLR* gene exhibit upregulation in porcine OECs cultivation. The last gene observed to be related to the ‘cell development’ GO term was *THY1*, also known as *CD90*. The *THY1* surface antigen has been described as a marker of gonocytes in the testicles of piglets [27]. Orsi et al. [28] also suggested that *THY1* was expressed on rat MSCs. In addition, *THY1* expression was also demonstrated in porcine neural stem cells and amniotic fluid-derived stem cells, which induced differentiation into oligodendrocytes, astrocytes, neurons, adipocytes, osteoblasts, myocytes and endothelial cells [29]. The *THY1* gene was upregulated, which could be associated with the potential for differentiation, making *THY1* a candidate surface marker for OEC in vitro differentiation.

The further described genes belong exclusively to the “cell differentiation” group, which may suggest their potential to be candidates as markers of this process in vitro. Herein, we have shown a group of genes which are mainly responsible for different metabolic processes in the cell: *SERPINB2, TXNIP, CRABP2, SLC5A1, ANPEP, FST*. All these genes showed down-regulated expression.

*SERPINB2*, also called plasminogen activator inhibitor type 2 [30], is one of the most upregulated proteins after cellular stress [31]. Decreased expression of this gene was also noted during culture of porcine OECs [6], while up-regulation was demonstrated upon in vitro culture of porcine granulosa cells [22].

Thioredoxin is a small oxidoreductase that plays an important role in antioxidative processes. *TXNIP* regulates cellular glucose metabolism by binding and inhibiting thioredoxin [32]. Salhab et al. [33] demonstrated that *TXNIP* was first expressed during oocyte in vitro maturation in bovine cumulus cells. The same study also suggested that *TXNIP* levels were lower in vitro than in vivo [33], which may lead to less effective energy supply in maturing oocytes in vitro, affecting their quality. Ożegowska et al. [34] determined the gene expression profile of cultured porcine oocytes, in which estrogen stimulation caused significant downregulation of *TXNIP* expression during in vitro maturation. A recently described study also showed down-regulation of this gene during primary culture of porcine granulosa cells [22,35]. In agreement with these previous studies, the present study demonstrated that the *TXNIP* gene was downregulated in cultured porcine OECs in vitro, which may suggest that *TXNIP* downregulation could serve as a marker for epithelial cell growth.

Another downregulated gene, *CRABP2*, belongs to a superfamily of lipid-binding proteins, which function to maintain tolerable intracellular retinoic acid concentrations [36]. A previous study carried out on mouse embryos at different stages of development demonstrated that both *CRABP1* and -2 transcripts were present in the embryo from the earliest stages and displayed specific distribution patterns [37].

The next gene, *SLC5A1*, was downregulated after in vitro culture of porcine OECs. This gene encodes a glucose transporter conjugated to sodium [38]. Ohta et al. [39] demonstrated that the expression of *SLC5A1* in the LLC-PK1 porcine kidney cell varied with the concentration of hexose metabolites (glucose) and cellular energy status.

Another gene involved in metabolic processes is *ANPEP*, which encodes a membrane-bound protein that catalyzes the formation of natriuretic IV hexapeptide angiotensin IV from angiotensin III [40]. It is mainly expressed in the kidney.

The last gene described in the context of metabolic processes is follistatin (*FST*), expressed in various tissues, especially in the ovaries, where it acts as an antagonist to activin [41]. *FST* inhibits the formation of XY gonad-specific coelomic vessels in XX gonads, which may be important in the early stages of ovarian development [42]. Ożegowska et al. [34] demonstrated that *FST* was downregulated after in vitro maturation of porcine oocytes, which may indicate that low *FST* expression could serve as a potential marker of in vitro maturation of oocytes [34]. Similarly, a significant decrease in *FST* gene expression was observed in OECs, which may emphasize its role as a marker of cellular maturation.

Several downregulated genes were molecules associated with immunological processes, including *CD274*, indoleamine 2,3-dioxygenase 1 (*IDO1*) and *DExD* box polypeptide 60 (*DDX60*). The first example of a gene from this group is the *CD274* gene, which encodes programmed death ligand-1 [43], *IDO1* contributes significantly to the immune response by participating in the metabolism of tryptophan [44]. The *DDX60* gene, which encodes an interferon-dependent helicase was also downregulated [45]. These three genes are involved in the process of immune response.

*CELA1* was yet another gene that showed downregulation after in vitro culture. This gene encodes the chymotrypsin-like elastase family member 1 protein, which belongs to a group of endopeptidases [46]. It is expressed in porcine ovaries, spleen and other tissues [47].

While genes with reduced expression belonged exclusively to the “cell differentiation” ontology group, genes that increased their expression showed different memberships.

The first example of a gene exclusively belonging to the “cell differentiation” group, characterized with strong change in expression, is *COL1A2*, encoding collagen type I. This protein is widespread in the animal organism and is most commonly found in subcutaneous adipose tissue [47]. Subsequently, high expression was also shown by the *LOX* gene, which is an enzyme that participates in the formation of precursors for collagen and elastin, and consequently supports the stabilization of collagen fibers [48]. Huang et al. [49] suggested that the *LOX* was an essential element for a normal epithelial-mesenchymal transition process, which was induced by bone morphogenetic protein 4 (BMP4). Another study, carried out on C3H10T1/2 cells treated with BMP4, indicated adipocyte lineage commitment after upregulation of *LOX* [50]. In the present study, *LOX* was upregulated, which could be related to the extracellular matrix formation processes, or potential differentiation of OECs into mesenchymal cells.

Subsequently, significant up-regulation of the *NOX4* gene, which also appears to be important in cell transdifferentiation, was also demonstrated. *NOX4* participates in the production of reactive oxygen species (ROS). *NOX4* may also contribute to the differentiation of fibroblasts into myofibroblasts, where it influences the induction of key myofibroblast characteristics, including contractility and matrix production [51]. Cucoranu et al. [52] suggested that *NOX4* plays a significant role in the conversion of fibroblasts into myofibroblasts, mediating the transforming growth factor-β1-induced differentiation of cardiac cells. Similar results were reported by Bondi et al. [53] in kidney fibroblasts. In addition, a previous study on cerebral microvascular endothelial cells (CMVEC) from newborn piglets indicated that *NOX4* protected CMVEC from tumor necrosis factor α-induced apoptosis, while another study suggested a role in the induction of cell death through ROS production [54,55]. A previous study carried out on porcine epithelial cell lines demonstrated that myocardin-related transcription factor, together with the state of the actin cytoskeleton, can regulate *NOX4* expression and *NOX4*-dependent ROS production, suggesting a relationship between the organization of the cytoskeleton and the cellular redox state [51]. In agreement with previous studies, *NOX4* was upregulated in the present study, suggesting a potential role of this gene as a marker of cell differentiation. In addition, the important role of *NOX4* in the redox state could indicate the advanced state of the maturational processes.

The next gene from the “cell differentiation” ontology group, expression of which was also increased after in vitro culture of OECs in the present study, was *UNC45B*. The *UNC45B* gene encodes asunc-45 myosin chaperone B. Using semi-quantitative reverse transcription PCR, Xu et al. [56] suggested that the porcine *UNC45B* gene was expressed exclusively in the striated muscle tissue and the heart. A previous study demonstrated that general cell (GC) *UNC45* expression was related to cell proliferation and cytoskeleton formation, while striated muscle (SM) *UNC45* expression mainly accompanied the formation of sarcomeres [57]. A previous study has reported that *UNC45B* functions during differentiation and muscle maturation processes [56]. Thus, it can be hypothesized that upregulation of the *UNC45B* gene in OECs in vitro may be a marker of cell proliferation, but also maturation, through the formation of the cytoskeleton or the involvement of myosin in the preparation for cell division.

In addition, *LIPG* upregulation was also demonstrated in the present study. *LIPG*, also known as endothelial lipase, is expressed in various tissues [47]. The expression of the *LIPG* gene has been demonstrated in a study on brain capillary endothelial cells, which are involved in the formation of the blood-brain barrier. This study indicated that *LIPG* can play a role in the formation of fatty acids and their passage through the barrier to the deeper areas of the brain, enabling the formation of cell membrane phospholipids [58]. Upregulation of *LIPG* in OECs can be a possible marker of cell maturation and proliferation, as the action of *LIPG* leads to the formation of cell membranes which allows for development of new cells.

*SGCE* is a gene encoding ɛ-sarcoglycan. It belongs to the imprinted gene group in pigs, leading to parent-specific monoallelic expression from either the paternal or maternal chromosome [59]. In the present study, this gene exhibited upregulation. Porcine ɛ-sarcoglycan is widely expressed in various tissues, including the ovaries and fat tissue [47], while in humans its expression is upregulated in the central nervous system [60]. *SCGCE* may be important in the transmission of impulses between neurons [61].

Metabolic processes taking place in cells preparing for differentiation or maturation require cytoplasmic changes, including reorganization of organelles and accumulation of RNA necessary for future protein synthesis. However, due to the complexity of these processes, further research is required to provide insight into the regulation of these processes. OEC in vitro gene expression profile was described, focusing particularly on the genes that regulate the aforementioned process. The present findings provide a basic molecular understanding of changes taking place in OECs in vitro, which both improves current knowledge concerning the possible ex vivo properties of these cells and could serve as a reference for further in vivo and clinical studies. Given the characteristic “dialogue” between cells of the reproductive system, including the interactions of the oviduct mucosa with the gamete during its journey, understanding the molecular basis of the processes that regulate important cellular life processes is essential. The identification of these genes could serve as a preliminary step towards the use of OECs in stem cell medicine.

## 4. Materials and Methods

### 4.1. Animals-Anatomical Structure and Collection of Oviducts

Local crossbred landrace gilts aged ~9 months (n = 45), which displayed two regular estrous cycles, were obtained at a commercial slaughterhouse. All the animals were checked daily for estrus behavior and were sacrificed after reaching the anestrus phase of the estrus cycle. The oviducts were excised within 25 min of slaughter. After cutting the abdominal wall in the linea alba, the reproductive system was removed along with the digestive tract. The oviducts were then collected to include: the tubal infundibulum, the ampulla of uterine tube and the isthmus of uterine tube, and transported to the laboratory within 30 min at 38 °C. Pig’s oviducts are between 12–25 cm. The wall of the oviducts consists of mucous membrane, muscular layer and serosa. Mucous membrane forms the least developed folds in the isthmus and much better in the remaining sections. As the mucosa folds develop, the muscular layer becomes thinner. Oocytes may be visible in the lumen of the oviduct. The use of animals for the purpose of scientific research complied with all the relevant national regulations and institutional policies for the care and use of animals. All experiments were performed on tissues from animals sacrificed in a commercial slaughterhouse. The authors did not participate in the procedure of sacrificing animals.

### 4.2. OEC Selection and Culture

The present study solely employed primary cultures of cells obtained from animal tissue. Oviducts were washed twice in PBS (137 mM NaCl; 27 mM KCl; 10 mM Na2HPO4; 2 mM KH2PO4; pH 7.4). Epithelial cells were surgically removed using sterile blades. Then, the epithelium was incubated with 1 mg/mL type I collagenase in DMEM (both from Sigma-Aldrich, St. Louis, MO, USA) for 1 h at 37 °C. The cell suspension obtained from this digestion was filtered through a 40-µm strainer to remove blood and aggregated cells. The remnant was collected by rinsing the strainer with DMEM, followed by centrifugation at 200× *g* for 10 min at room temperature (RT), as previously described [62,63]. Next, the resulting pellet was washed in PBS and centrifuged again at 200× *g* for 10 min at RT. Cells were then incubated with 0.5% Trypsin/EDTA (Sigma-Aldrich St. Louis, MO, USA) at 37 °C for 10 min. The reaction was stopped with fetal calf serum (FCS; Sigma-Aldrich, St. Louis, MO, USA). After incubation, cells were filtered and centrifuged again at 200× *g* for 10 min at RT. The final cell pellet was resuspended in DMEM supplemented with 10% FCS, 100 U/mL penicillin, 100 µg/mL streptomycin and 1 µg/mL amphotericin B. The cells were cultured at 37 °C in a humidified atmosphere of 5% CO2. Once the OEC cultures attained 70–80% confluency, they were passaged by washing with PBS, digested with 0.025% Trypsin/EDTA for 3–6 min at 37 °C, neutralized by a 0.0125% trypsin inhibitor (Sigma-Aldrich, St. Louis, MO, USA ), centrifuged at 200× *g* for 10 min at RT and resuspended at a seeding density of 2 × 10^4^ cells/cm^2^. The culture medium was changed every three days. The culture lasted 30 days.

### 4.3. Hematoxylin and Eosin (H&E) Staining

Immediately after collection, ten oviducts were fixed with Bouin’s solution (a mixture of picric acid, 40% formaldehyde and acetic acid; picric acid and acetic acid were mixed 15:1) for 48 h at RT. Subsequently, tissues were embedded in paraffin wax and cut into 3–4 μm sections with a semi-automatic rotary microtome (Leica RM 2145; Leica Microsystems GmbH, Nussloch, Germany). Oviductal sections were deparaffinized and rehydrated prior to H&E staining, and finally dehydrated. The sections were stained in hematoxylin for 20 min and in eosin for 40 min at RT. The sections were observed with a light microscope and selected images were taken with a high-resolution scanning technique using an Olympus BX61VS microscope scanner (Olympus Corporation, Tokyo, Japan). Slides were scanned at magnification, ×20. The images presented in publication consist of digitally magnified selected fragments of the scans.

### 4.4. RNA Extraction from Porcine OECs

OECs investigated at each time period were pooled into three independent samples for each culture time intervals. Total RNA was extracted from samples using TRI Reagent (Sigma-Aldrich; St. Louis, MO, USA) and RNeasy MinElute Cleanup kit (Qiagen GmbH, Hilden, Germany). The amount of total mRNA was determined from measuring the optical density (OD) at 260 nm, and RNA purity was estimated using the 260/280 nm absorption ratio on a NanoDrop™ spectrophotometer (Thermo Fisher Scientific, Inc., Waltham, MA, USA). Samples were considered pure if the ratio was >1.8. RNA integrity and quality were checked on a Bioanalyzer 2100 (Agilent Technologies, Inc., Santa Clara, CA, USA). The resulting RNA integrity numbers were between 8.5 and 10, with an average of 9.2. The RNA in each sample was diluted to a concentration of 100 ng/μL with an OD260/OD280 ratio of 1.8–2.0. For each sample, 500 ng RNA was taken for the microarray analysis.

### 4.5. Microarray Expression Analysis

RNA (100 ng) extracted from samples from all of the investigated intervals (1, 7, 15 and 30 days of culture) was subjected to two rounds of sense cDNA amplification (first round: 5 min incubation at 42 °C, followed by chilling on ice for 1 min, second round: 15 min incubation at 42 °C with reaction stopped by heating at 95 °C for 5 min) using a WT Expression kit (Ambion; Thermo Fisher Scientific, Inc., Waltham, MA, USA). The obtained cDNA was used for biotin labeling and fragmentation by Affymetrix GeneChip^®^ WT Terminal Labeling and Hybridization. Biotin-labeled fragments of cDNA (5.5 μg) were hybridized to the Affymetrix Porcine Gene 1.1 ST Array Strip at 48 °C for 20 h. Microarrays were then washed and stained according to the technical protocol using the Affymetrix GeneAtlas™ Fluidics Station. The array strips were scanned using the Imaging Station of the GeneAtlas System (Affymetrix; Thermo Fisher Scientific, Inc., Santa Clara, CA, USA). Preliminary analysis of the scanned chips was performed using Affymetrix GeneAtlas Operating Software (version 2.0.0.460; Affymetrix; Thermo Fisher Scientific, Inc., Waltham, MA, USA). The quality of gene expression data was confirmed according to the quality control criteria provided by the software. The obtained CEL files were imported into downstream data analysis software. All microarray instrumentation and software were from Affymetrix (Thermo Fisher Scientific, Inc., Waltham, MA, USA).

### 4.6. Real-Time Quantitative Polymerase Chain Reaction (RT-qPCR) Analysis

Transcript level examination of 20 selected genes from GO BP were performed using a Light Cycler^®^ 96 Real-Time PCR System, Roche Diagnostics GmbH (Mannheim, Germany). The dye used for detection was QUANTUM Eva Green PCR Kit (Syngen Biotech, Wroclaw, Poland). The study was carried out in the time intervals assumed for the analysis of the transcriptome of OEC cells. The selected 20 genes were validated in 3 biological repetitions. RNA samples were treated with DNAse I and reverse transcribed (RT) into cDNA. For each RT reaction, 200 ng of RNA transcript was used. Primer3Plus software (version 0.4.0; Whitehead Institute for Biomedical Research, Massachusetts Institute of Technology, Cambridge, MA, USA) has been used to design primers (Table 2).

The exon-exon design method avoided possible amplification of genomic DNA fragments. Primers were also designed using the sequences of several transcript variants of selected genes available in the Ensembl database. The electrophoresis of the products on a 2% agarose gel, as well as the analysis of dissociation curves in the software of the equipment used, allowed to confirm the specificity of the obtained results.

The reaction mixture of RT-qPCR consisted of: 1 µL cDNA, 6 µL QUANTUM Eva Green PCR Kit (Syngen Biotech), 3 µL nucleas free water. Thermocycling conditions were as follows: Preincubation at 37 °C for 30 s; 3-step amplification (95 °C for 15 s, 58 °C for 15 s, 72 °C for 15 s) for 40 cycles; melting (95 °C for 60 s, 40 °C for 60 s, 70 °C for 1 s, 95 °C for 1 s); cooling at 37 °C for 30 s. Transcript levels in each sample were analyzed against *β-actin (ACTB)* and *glyceraldehyde 3-phosphate dehydrogenase (GAPDH*) as internal controls. For target cDNA quantification, we have performed relative quantification with the 2 − ΔΔCT method [64].

### 4.7. Bioinformatics and Statistical Analysis

The present analysis was performed using Bioconductor (version 3.11; http://www.bioconductor.org/) and R (version 3.5.1; www.r-project.org) programming packages. Each CEL file was merged with a description file. In order to correct background, normalize and summarize results, the Robust Multiarray Averaging algorithm was used [65]. To determine the statistical significance of the analyzed genes, moderated t-statistics from the empirical Bayes method were performed. The obtained *p*-value was corrected for multiple comparisons using Benjamini and Hochberg’s false discovery rate. The selection of significantly altered genes was based on corrected *p* < 0.05 and fold change in expression >|2| [66].

Differentially expressed genes were selected based on their involvement in cell differentiation, maturation, development and growth. The differentially expressed gene list was uploaded to the Database for Annotation, Visualization and Integrated Discovery (DAVID; version 6.8; DAVID) [67] to obtain GO terms and Kyoto Encyclopedia of Genes and Genomes (KEGG) pathways. Expression data of these genes were subjected to hierarchical clustering, with their expression values presented as heatmaps, as in previous studies [68] using Pathview software (version 3.11; Bioconductor) [69]. Pathview is a tool set for pathway-based data integration and visualization. It maps and renders a wide variety of biological data on relevant pathway graphs.

The relation between the genes belonging to chosen GO terms and KEGG pathways were then analyzed using the GOplot software, (version 3.11; Bioconductor, open source project; http://www.bioconductor.org/) [70]. The GOplot package calculate the z-score as (the number of upregulated genes–the number of downregulated genes)/√count. This information allowed the estimation of direction of total expression changes in each of the GO terms and KEGG pathways.

Interactions between differentially expressed genes/proteins belonging to the studied GO group were investigated using Search Tool for the Retrieval of Interacting Genes (STRING) 10 [71]. The list of gene names was used as a query for an interaction prediction. The search criteria were based on co-occurrences of genes/proteins in scientific texts (text mining), co-expression and experimentally observed interactions, resulting in a gene/protein interaction network where the intensity of the edges represents the strength of the interaction score.

Finally, the functional interaction (FI) between genes that belongs to the chosen GO biological process (BP) terms were investigated using the Reactome FIViz application in the Cytoscape 3.6.0 software (https://cytoscape.org/), as described in our previous studies [72]. The ReactomeFIViz application is designed to find pathways and network patterns related to cancer and other types of diseases. This application accesses the pathways stored in the Reactome database, which allows for pathway enrichment analysis to be performed for a set of genes, hit pathways can be visualized using manually laid-out pathway diagrams directly in Cytoscape, and functional relationships among genes in hit pathways can be investigated. It can also access the Reactome Functional Interaction network, a highly reliable, manually curated pathway-based protein FI network covering >60% of human proteins.

### 4.8. Nano Liquid Chromatography (LC)-MALDI-TOF/TOF MS/MS Analysis

Three purified samples were fractionated using nanoLC: EASY-nano LC II connected with a Proteineer-fc II fraction collector (Bruker Daltonics, Billerica, MA, USA). Detailed conditions of the performed separation, as well as specifications of LC columns were described in our previous research [73]. A total of 384 fractions were obtained, each automatically mixed with HCCA matrix solution and spotted onto MALDI target plate (MTP AnchorChip 384, Bruker Daltonics, Billerica, MA, USA). Subsequently, MALDI-TOF/TOF MS/MS analysis was performed using the UltrafleXtreme instrument. Typical settings of the apparatus for MS and MS/MS mode were described in our previous study [73]. MS spectra were externally calibrated with the use of peptide calibration mixture in the 0.7–3.5 kDa mass range. WARP-LC 1.3 platform was used to select precursor ions with signal-to-noise threshold above 5 for MS/MS analysis. The following platforms and software were used for analysis, data acquisition and evaluation: WARP LC 1.3, ProteinScape 3.1, FlexControl 3.4 and FlexAnalysis 3.4. All instruments and software were from Bruker Daltonics (Bruker Corporation, Billerica, MA, USA).

Protein search was conducted using the NCBI database with the Mascot 2.4.1 search engine (Matrix Science, London, UK) using the following parameters: (i) Trypsin digestion; (ii) missed cleavage, 1; (iii) peptide mass tolerance, 25 ppm; (iv) MS/MS fragment mass tolerance, 0.7 Da; (v) peptide charge, 1+; (vi) monoisotopic mass; and (vii) carbamidomethylation of cysteine as fixed modification. The searches were restricted to ‘other mammals’. Only proteins with at least two unique peptides, score > 80 and false discovery rate < 1% were included. Results obtained from three analysis were combined into one list of proteins using ProteinScape 3.1. software.

## Figures and Tables

**Table 1:** Gene symbols, fold changes in expression, Entrez gene IDs and corrected *p* values of studied genes.

Gene Symbol	Fold Change D7/D1	Fold Change D15/D1	Fold Change D30/D1	Adjusted *p*-Value D7/D1	Adjusted *p*-Value D15/D1	Adjusted *p*-Value D30/D1
*SERPINB2*	0.041199	0.012677	0.008239	4.60 × 10^−6^	7.45 × 10^−7^	2.31 × 10^−7^
*CD274*	0.04424	0.04443	0.041834	2.50 × 10^−6^	7.45 × 10^−7^	2.31 × 10^−7^
*TXNIP*	0.082179	0.64453	0.63523	1.11 × 10^−5^	0.024019	0.017645
*CELA1*	0.10027	0.111224	0.091977	4.67 × 10^−5^	3.35 × 10^−5^	1.41 × 10^−5^
*DDX60*	0.104831	0.194221	0.213218	7.61 × 10^−5^	0.000244	0.000232
*CRABP2*	0.108046	0.084638	0.056801	3.11 × 10^−5^	1.05 × 10^−5^	2.84 × 10^−6^
*SLC5A1*	0.122502	0.081281	0.097509	3.30 × 10^−5^	7.64 × 10^−6^	6.67 × 10^−6^
*IDO1*	0.134544	0.12111	0.123331	7.22 × 10^−5^	3.53 × 10^−5^	2.36 × 10^−5^
*ANPEP*	0.143464	0.201711	0.482675	1.74 × 10^−5^	1.96 × 10^−5^	0.000794
*FST*	0.148055	0.075627	0.078555	1.84 × 10^−5^	2.99 × 10^−6^	1.32 × 10^−6^
*UNC45B*	10.72559	2.574362	1.464151	1.45 × 10^−5^	0.000645	0.040563
*NOX4*	11.05623	21.12763	32.07053	4.90 × 10^−6^	9.73 × 10^−7^	2.31 × 10^−7^
*VLDLR*	11.18756	15.02697	21.33103	1.03 × 10^−5^	3.25 × 10^−6^	8.85 × 10^−7^
*ITGB3*	12.67365	13.15962	10.9347	4.27 × 10^−6^	1.55 × 10^−6^	8.03 × 10^−7^
*FMOD*	12.87587	41.88541	41.90401	2.50 × 10^−6^	6.02 × 10^−7^	2.31 × 10^−7^
*SGCE*	13.222	29.18235	46.77017	5.88 × 10^−6^	9.76 × 10^−7^	2.51 × 10^−7^
*COL1A2*	15.57291	56.37555	61.32906	3.63 × 10^−6^	7.45 × 10^−7^	2.31 × 10^−7^
*LOX*	29.0517	43.84613	46.65297	7.88 × 10^−6^	2.86 × 10^−6^	1.06 × 10^−6^
*LIPG*	36.5469	19.67815	39.01801	2.50 × 10^−6^	1.39 × 10^−6^	2.57 × 10^−7^
*THY1*	38.75283	32.74287	31.30186	3.01 × 10^−6^	1.39 × 10^−6^	5.59 × 10^−7^

**Table 2:** Oligonucleotide sequences of primers used for RT-qPCR analysis.

Gene	Gene ID	Primer Sequence (5′–3′)	Product Size (bp)
*THY1*	100271931	CCAAAGATGAGGGGATCTACG CCAAAGATGAGGGGATCTACG	100
*LIPG*	100155736	ATCCTGAGAACACCCGCATA AGGATGCTCCACAGTTGGAC	104
*LOX*	100525278	CCAGAGGAGAGTGGCTGAAG CTGGGGTTCACACTGACCTT	216
*COL1A2*	100626716	GTCAGACTGGTCCTGCTGGT GTCAGACTGGTCCTGCTGGT	163
*SGCE*	100240725	CCAACAATCATTGAGATAACTGC GCTGGCCAACATTTCTTCTA	156
*FMOD*	100526237	TGCTCACTGGGTCTGTGAAG CCTCAAAGATAGGGGCTTCC	194
*ITGB3*	397063	GGCTTCAAAGACAGCCTCAC AGTCCTTTTCCGAGCACTCA	175
*VLDLR*	733630	TGAGCCTTCCCAATTCCAGT CATATGGCACTGTTCTGGGC	232
*NOX4*	100523323	ACAACTGTTCCTGGCCTGAC CAGCCCTCCTGAAACATGTAA	168
*UNC45B*	100134956	GCCTGAAAACGGAGAGCTATG ACAACGCTGCACGTCCTT	152
*FST*	445002	CTGAGCACCTCCGACGAG ACGTTTCTTTACATGGGATGC	100
*ANPEP*	397520	CCACCATCTACTGCAATGCC CGTCTTGCTTCCGAATGAGG	195
*IDO1*	100519877	CACTGTGGGTGGAGTTCCTT TTTCGCAGGGATACCATAGC	198
*SLC5A1*	397113	CTTTGCCATCATCCTCTTTG CCAACACAGGCGGTAGAGAT	100
*CRABP2*	100155151	GAGCCTGGTAAAATGGGAGA CTCGGACATAGACCCTGGTG	164
*DDX60*	100158037	GCTCATGCTCCTGGCTTC CTTGACCAGGAATTGCAGTG	154
*CELA1*	396766	GCTCTGGATGTCAGGGTGAT GCTCTGGATGTCAGGGTGAT	176
*TXNIP*	733688	CAAGCCAGCCAACTCAAGAG TTCGAGCAGAGACAGACACC	212
*CD274*	574058	GTGTTGGTCATCCCAGAACC TATCTCGGCTGCCACATTTT	157
*SERPINB2*	100519286	GGAAGAATACATTCGACTCTCCA GGAAGAATACATTCGACTCTCCA	170

## Data Availability

Not applicable.
